# Biomechanical evaluation and comparison of clinically relevant versus non-relevant leg length inequalities

**DOI:** 10.1186/s12891-022-05113-2

**Published:** 2022-02-23

**Authors:** Roman Michalik, Viola Rissel, Filippo Migliorini, Hannah Lena Siebers, Marcel Betsch

**Affiliations:** 1 Department of Orthopedic, Trauma and Reconstructive Surgery, University Hospital RWTH Aachen, Pauwelsstraße 30, 52074 Aachen, Germany; 2 Department of Orthopedic Surgery, University Hospital RWTH Aachen, Aachen, Germany; 3 Department of Orthopedic Surgery, University of Toronto, Toronto, Canada; 4 Department of Orthopaedics and Trauma Surgery, Medical Faculty of the University Heidelberg, University Hospital Mannheim, Mannheim, Germany

**Keywords:** Leg length inequalities, Leg length discrepancy, Rasterstereography, Gait, Surface topography

## Abstract

**Background:**

Leg length inequalities are a frequent condition in every population. It is common clinical practice to consider LLIs of 2 cm and more as relevant and to treat those. However, the amount of LLIs that need treatment is not clearly defined in literature and the effect of real LLIs on the musculoskeletal system above and below 2 cm have not been studied biomechanically before.

**Methods:**

By using surface topography, we evaluated 32 patients (10 females, 22 male) with real LLIs of ≥ 2 cm (mean: 2.72 cm; *n* = 10) and compared their pelvic position and spinal posture to patients with LLIs < 2 cm (mean: 1.24 cm; *n* = 22) while standing and walking. All patients were measured with a surface topography system during standing and while walking on a treadmill. To compare patient groups, we used Student t-tests for independent samples.

**Results:**

Pelvic obliquity was significantly higher in patients with LLI ≥ 2 cm during the standing trial (*p* = 0.045) and during the midstance phase of the longer leg (*p* = 0.023) while walking. Further measurements did not reveal any significant differences (*p* = 0.06–0.706).

**Conclusions:**

The results of our study suggest that relevant LLIs of ≥ 2 cm mostly affect pelvic obliquity and do not lead to significant alterations in the spinal posture during a standing trial. Additionally, we demonstrated that LLIs are better compensated when walking, showing almost no significant differences in pelvic and spinal posture between patients with LLIs smaller and greater than 2 cm. This study shows that LLIs ≥ 2 cm can still be compensated; however, we do not know if the compensation mechanisms may lead to long-term clinical pathologies.

## Background

Leg length inequalities are a common finding in every society with a prevalence of up to 90 percent. [1, 2]. For decades research has been published, defining the amount of LLI that needs to be treated; however, a clear consensus in the medical community has not been reached yet [1, 3–8]. In early work by Gross et al. in 1978, it was concluded that “there seems little indication for equalization of discrepancies less than 2 cm” [9], while treatment above this amount needs to be decided individually as relevant clinical and biomechanical data is missing. Biomechanical studies have suggested that LLIs of 2 cm and more lead to relevant changes in the knee and ankle joints as well as to pelvic obliquity resulting in gait asymmetry [4, 5, 10]. Based on these findings some authors suggest to treat LLIs of more than 2 cm, while others recommend even earlier interventions [1–3, 6, 7]. Therefore, today LLIs of 2 cm and greater are widely considered clinically relevant and do warrant treatment, however without clear evidence in literature [8].

Several studies have evaluated the temporary and permanent changes in pelvic position and spinal posture caused by LLIs [1, 11–14], which may cause musculoskeletal disorders, such as lower back pain, functional scoliosis, gait disorders and osteoarthritis of the hip and knee joints [3, 15, 16]. However, the direct clinical effects and consequences of LLIs do vary between individuals and not all clinical symptoms do directly correlate with the amount of leg length asymmetry [1, 2, 17]. Nonetheless, it seems recommended to treat LLIs that do exceed compensation mechanisms and lead to significant changes in pelvic position and spinal posture.

We consider it necessary to evaluate the clinically relevant amount of LLI in an objective and biomechanical setting. Therefore, purpose of this study was to examine and compare the effects of patients with LLIs < 2 cm with patients with LLIs ≥ 2 cm. We hypothesize that LLI ≥ 2 cm lead to significantly greater effects on pelvic position and spinal posture.

## Material and methods

### Human subjects

A sample size estimation (80% power, level of significance 5%) revealed that 30 participants were to be included in this study for sufficient statistical power (Software G*Power, Version 3.1, HHU Düsseldorf, Germany).

Included were 32 adults (10 females, 22 male) with structural LLIs greater 1 cm. Twenty-one patients had congenital LLIs, while acquired LLIs were caused by total hip replacement (*n* = 6), total knee and hip replacement (*n* = 3), Perthes disease (*n* = 1) or previous fractures of the lower leg (*n* = 1).

We excluded patients with acute injuries or pain of the spine, pelvis or lower extremities as well as obesity with a body mass index (BMI) > 35 kg/m^2^. The study protocol was approved by the local ethic committee (Ethics Committee at the RWTH Aachen Faculty of Medicine, EK 111/15), and there was compliance with the principles of the seventh revision of the Declaration of Helsinki, as well as the Good Clinical Practice Guidelines throughout the study.

For the purpose of this study, we divided the overall cohort in patients with LLI < 2 cm (mean = 1.24 cm; *n* = 22) and LLI ≥ 2 cm (mean = 2.72 cm; *n* = 10). Overall, the were no differences between the two groups in terms of their demographic data (Table 1).

**Table 1:** Demographic data of patient groups used in this study. Patients were distributed in two groups with structural LLIs < 2 cm (Group A) and ≥ 2 cm (Group B). The table presents mean value of demographic data and standard deviation

	**Group A**	**Group B**	***p*-value**
n	22	10	
LLI (cm)	1.24 ± 0.2	2.72 ± 1.18	0.003
Age (years)	44 ± 21	48 ± 20	0.647
Weight (kg)	79 ± 12	80 ± 14	0.743
Height (cm)	175 ± 0.009	179 ± 0.12	0.367
BMI	25.58 ± 3.77	25.04 ± 3.77	0.709

### Measurement protocol

Patients were clinically examined and the leg length and their differences was measured with a tape from the anterior superior iliac spine to the tip of the medial malleolus [18], which has been proven to be highly valid and reliable compared to CT measurements [19–21]. The measurement protocol used was described previously [12]. In our study, we evaluated pelvic posture and spine with a surface topography system (DIERS 4D motion ® Lab, Diers International GmbH, Schlangenbad, Germany), which consists of a multi-camera system and a treadmill that was used for the dynamic measurements. This setup does allow to evaluate gait phases, which can be synchronized with the respective pelvic position and spinal posture [11–13, 22].

First, static measurements were conducted with the patients standing in an upright position with extended knees and arms hanging on the sides in a neutral standing position. All relevant parameters that were measured described in the section below.

Secondly, for the dynamic measurements all patients were walking for 30 s with a velocity of 3 km/h barefoot on the treadmill and values measured were averaged over this time. Gait phases were analyzed based on the gait phases described by Perry et al. and previous work [12, 23, 24]. The stance phase of each leg was subdivided into initial contact of the foot touching the ground (t0), mid-stance when the whole foot has contact (t1) and the terminal contact phase before the foot lifts off the ground (t2).

### Surface topography

To image and analyze the patients under static conditions and while walking we used radiation free surface topography. The system has been used previously and it has shown its validity and reliability in multiple studies [25–28].

In patients with scoliosis Knott et al. evaluated the inter- class corelation coefficient (ICC) for kyphotic Angle (0.984) lordotic Angle (0.977) and pelvic obliquity (0.894) [29].

Abdel Raoof et al. evaluated exclusively pelvic parameters for their reliability and validity on healthy subjects. ICCs for inter-/intra examiner reliability and validity were checked for pelvic torsion (0.99/ 0.999), pelvic tilt (0.997/0.999) and pelvic inclination (0.989/0.998). Validity to radiography evaluated with pearsons corelation coefficient (r) for pelvic torsion (*r* = 0.867), pelvic tilting (*r* = 0.996) and pelvic inclination (*r* = 0.930) [30].

Intraday reliability (using ICC) for surface rotation to the left (0.832) and right (0.797) as well as lateral deviation to left (0.772) and right (0.829) were evaluated by Guidetti el al.. [25].

A meta-analysis by Krott et al. regarding reliability and validity of surface topographic measurements on patients concluded that it shows satisfactory reliability and validity in detecting spinal posture [28].

**Fig. 1:**
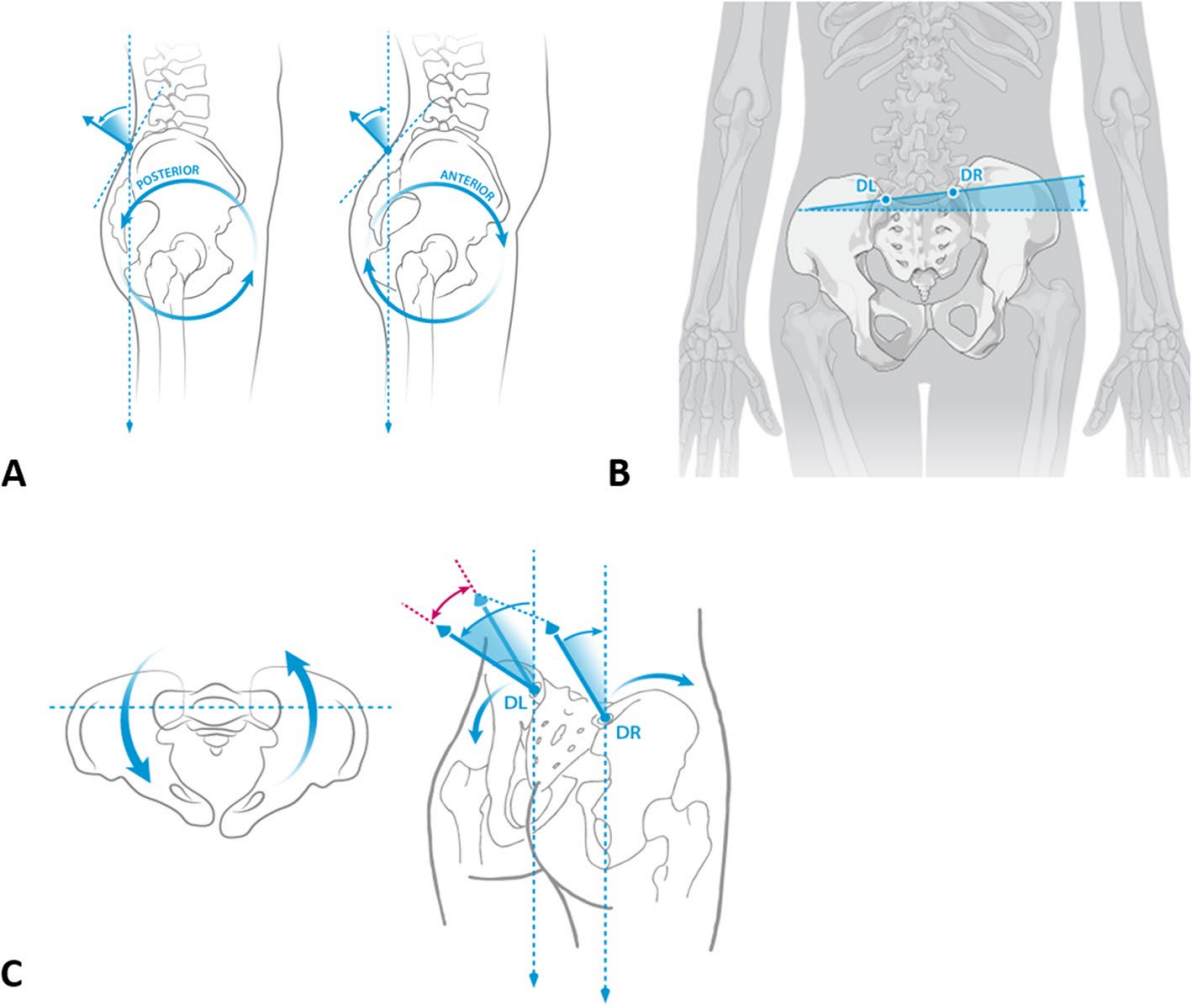
The following pelvic parameters were measured using surface topography: pelvic inclination is defined as the mean vertical torsion of the two surface normals of the lumbar dimples **A**. Pelvic obliquity is the amount of tilt of the right (DR) and the left lumbar dimple (DL) from a horizontal line **B**. Pelvic torsion is the rotation of DL and DR to each other measured in degrees **C** (Images used with kind permission of Diers International GmbH)

The following pelvic and spinal parameters were evaluated in this study and are briefly described here and in previous work [11–13, 27] (Fig. 1 A-C):

Pelvic obliquity is the amount of tilt of the right (DR) and the left lumbar dimple (DL) from a horizontal line measured in millimeters (Table 2). Pelvic torsion is the rotation of DL and DR to each other measured in degrees. The third pelvic parameter evaluated is the pelvic inclination which is defined as the mean vertical torsion of the two surface normals of the lumbar dimples.

**Table 2:** Pelvic parameters measured and their interpretation

Parameter	Positive value	Negative value
Pelvic obliquity (mm)	DR higher	DL higher
Pelvic torsion (°)	DR further anterior	DL further anterior
Pelvic inclination (°)	Anterior inclination	Posterior inclination

**Fig. 2:**
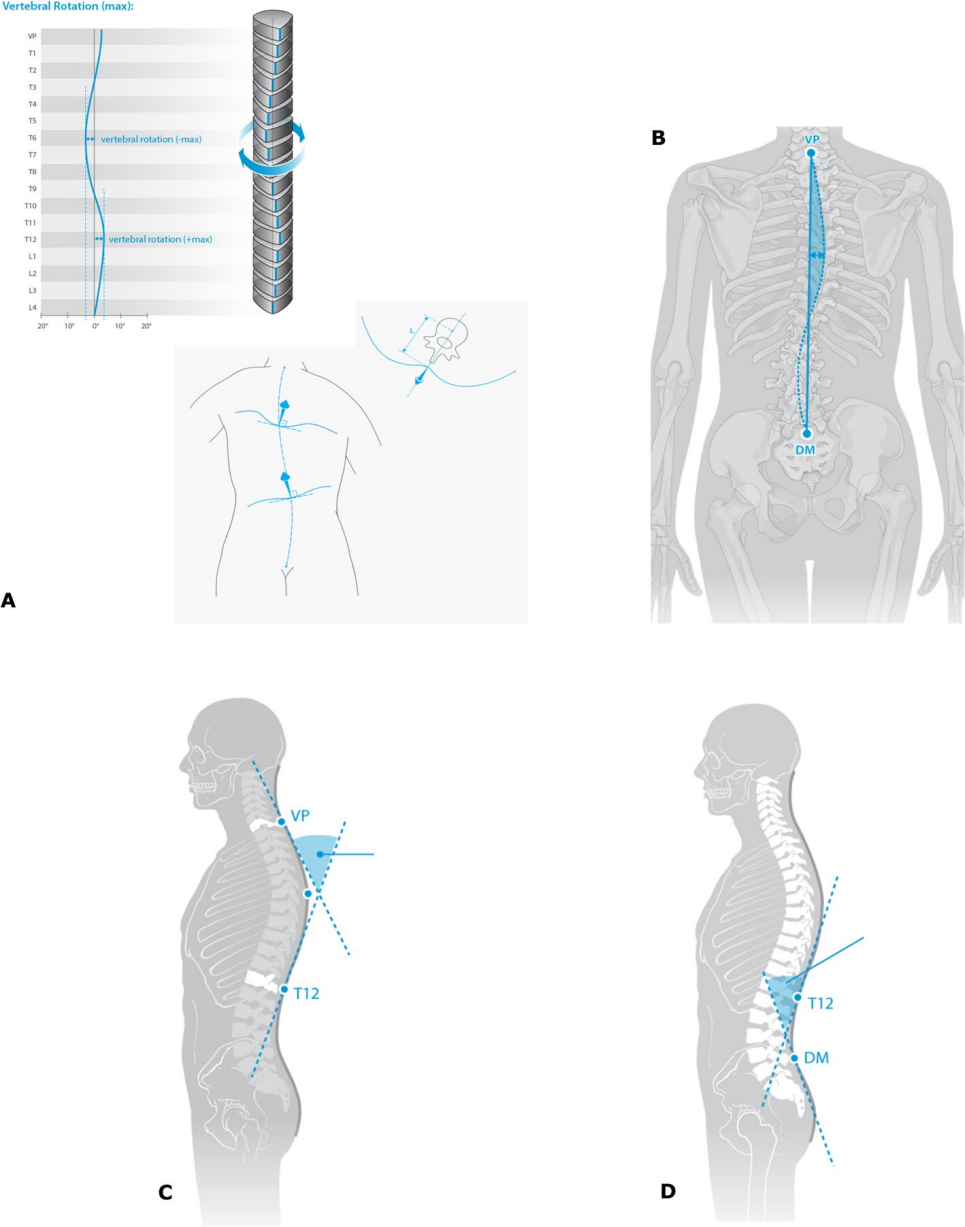
The surface rotation of the spine is the value of the horizontal components of the surface normals on the line connecting the spinous processes of the vertebrae **A**. Lateral deviation of the spinal midline (from spinal process of the 7th cervical vertebra to a midpoint between DL and DR) is measured and calculated as root mean square in the frontal plane **B**. Kyphotic angle is measured between the surface tangents on points VP (vertebra prominence) and the calculated spinous process of the 12th thoracic vertebrae (T12) **C**. Lordotic angle is the angle between the surface tangents on points T12 and the midpoint (DM) between DL and DR **D**. (Images used with kind permission of Diers International GmbH)

The surface rotation of the spine is the value of the horizontal components of the surface normals on the line connecting the spinous processes of the vertebrae (Fig. 2 A). It is calculated as root mean square in degrees. Lateral deviation is the root mean square of the deviation of the spinal midline (from spinal process of the 7th cervical vertebra to a midpoint between DL and DR) in the frontal plane (Fig. 2B). It is measured in millimeters. Kyphotic angle is the angle between the surface tangents on points VP (vertebra prominence) and the calculated spinous process of the 12th thoracic vertebrae (T12) (Fig. 2C).

Lordotic angle is the angle between the surface tangents on points T12 and the midpoint between DL and DR. Both angles are measured in degrees (Fig. 2D).

### Statistical analysis

For the statistical analysis patients were grouped in patients with LLIs < 2 cm and LLIs ≥ 2 cm.

For further calculations the LLIs were evaluated as side independent. Side specific data of patients with a longer left leg were multiplied by -1, while data of patients with a longer right leg were not changed. The terms of a longer ( +) and a shorter (-) leg were used.

To compare patient groups, we used Student t-tests for independent samples. Based on the results of Levene-test for variance-homogeneity we used either Students t-test or Welch-test for further analysis. The level of significance was set at p < 0.05. Statistical analysis was performed using SPSS software (IBM SPSS Statistics, Version 24, Chicago, IL, USA).

## Results

### Static measurements

**Fig. 3:**
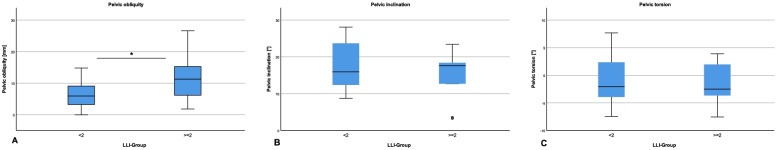
Boxplot graphics of measured pelvic parameters showing significant higher pelvic obliquity **A** in group B (LLI ≥ 2 cm). Pelvic inclination was smaller in the group with higher LLI **B**. This difference between groups, however was not significant (*p* = 0.35). For pelvic torsion the results showed no significant differences in neutral standing (*p* = 0.79). The negative value indicates that the left dimple is orientated further anterior **C**

Here, we compared the two groups regarding their differences in spinal and pelvic parameters during neutral standing. The results show, as expected, a significant higher pelvic obliquity (*p* = 0.05) in patients with LLIs ≥ 2 cm (group B) (Fig. 3A). However, there were no other significant differences found between the groups for pelvic torsion and inclination (*p* = 0.11–0.79) (Fig. 3B-C).

Besides pelvic parameters, we evaluated the influence of LLIs on the spinal posture. The results show a trend of increasing surface rotation and lateral deviation in patients with higher LLI (group B). However, the difference between groups was not statistically significant (Table 3).

**Table 3:** Results for static evaluation of pelvic and spinal parameters in patients with LLI < 2 cm (group A) and LLI ≥ 2 cm (group B)

	**Group A**		**Group B**		***p*-value**
	mean	SD	mean	SD	
**Pelvic Obliquity (mm)**	6.50	3.92	12.28	7.64	**0.045**
**Pelvic Torsion (°)**	-1.54	3.78	-1.93	3.71	0.79
**Pelvic Inclination (°)**	17.72	6.15	15.40	7.03	0.35
**Kyphotic Angle (°)**	54.12	7.30	51.41	14.15	0.58
**Lordotic Angle (°)**	37.46	6.24	34.91	9.01	0.36
**Surface rotation (°)**	3.71	1.87	3.92	1.96	0.78
**Lateral deviation (mm)**	4.06	2.82	5.91	3.26	0.11

A trend for a higher kyphotic angle was found in group A (54.12°) compared to group B with LLI ≥ 2 cm (51.41°), which was not significant (*p* = 0.58). Measurements of the lordotic angle showed similar results (*p* = 0.36) with a trend towards higher lordotic angles in the group with smaller LLIs.

### Dynamic measurements

We also analyzed and compared the effects of different LLIs on pelvic position and spinal posture during walking on a treadmill.

### Pelvic obliquity

**Fig. 4:**
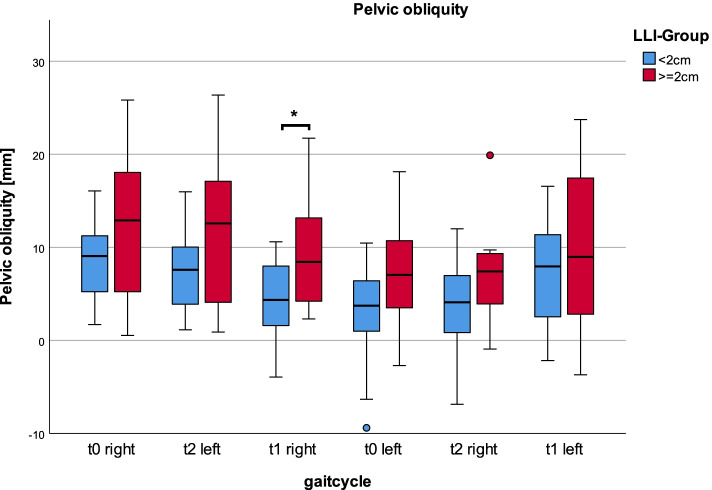
Boxplot graphics of measured pelvic obliquity in subjects with LLI < 2 cm (blue) and LLI ≥ 2 cm (red) while walking on a treadmill. Gait phases (t0: initial contact, t1: mid-stance, t2: terminal contact) of one gait cycle are listed on the x-axes. A significant (*) higher pelvic obliquity was shown in the t1 phase of the right (longer) leg (*p* = 0.023)

The pelvic obliquity was constantly higher in group B, which was significant during the t1 phase of the longer leg (*p* = 0.023). The highest value for pelvic obliquity was found in the initial contact phase of the longer leg (*p* = 0.252). Lower values were found during the midstance and terminal contact phase (*p* = 0.121) of the longer leg (Fig. 4).

### Spinal parameters

**Fig. 5:**
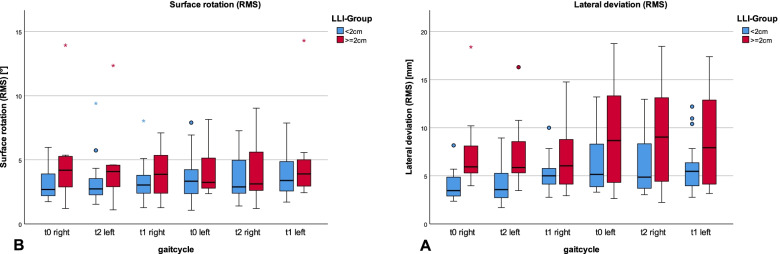
Boxplot graphics shows mean lateral deviation calculated as the root mean square while walking **A** and surface rotation **B** in subjects with LLI < 2 cm (blue) and LLI ≥ 2 cm (red). Stance phases of one gait cycle are listed on the x-axes: initial contact of the foot touching the ground (t0), mid-stance when the whole foot has contact (t1) and the terminal contact phase before the foot lifts off the ground (t2). Lateral deviation and surface rotation show orientation to the longer leg, which is indicated by positive values

The kyphotic angle was not significantly different between both groups during all gait phases (*p* = 0.451- 0.92). Also both groups showed similar lordotic angles while walking (*p* = 0.526–0.706). The analysis of the lateral deviation of the spine revealed a trend towards a higher lateral deviation in the group with LLIs ≥ 2 cm throughout the gait cycle, which was not statistically significant (*p* = 0.060–0.263) (Fig. 5A).

The investigation of surface rotation of the spine showed similar results. The rotation of the spine was orientated towards the longer leg (positive value). The rotation trended higher values in group B with the highest differences between groups in the initial contact phase of the longer leg (*p* = 0.065) (Fig. 5B). In all other gait phases no significant differences between the groups were found (*p* = 0.065–0.549).

## Discussion

So far, LLIs of 2 cm and greater are considered clinically relevant since they can lead to acute or chronic musculoskeletal changes and clinical symptoms [1, 8]. However, there still exist some controversies in the literature regarding the exact amount of LLI that is considered to be clinically significant and which needs to be treated [1, 3–6, 14]. Purpose of this study was to compare pelvic position and spinal posture in patients with LLI < 2 cm versus patients with LLIs > 2 cm during standing and while walking. The hypothesis was that patients with LLIs > 2 cm will have significantly altered pelvic position and spinal posture compared to patients with LLIs < 2 cm due to their larger LLIs.

The results of this present study show that patients with LLIs > 2 cm presented only with significant greater pelvic obliquity and not with any other pelvic parameter compared to patients with LLIs < 2 cm. In addition, no differences in spinal posture were found between the two groups. During our walking trials, when comparing pelvic position and spinal posture between the groups, our analysis found mostly no differences between the LLIs > 2 cm and the LLIs < 2 cm groups, indicating that LLIs are even more compensated during walking than while standing.

The effects of LLIs on the patient’s pelvis and spine while standing are supported by the current literature. Pelvic obliquity increases with increasing LLIs on the side of the longer leg as previously confirmed in real and simulated LLIs [4, 12, 13]. In addition, previously it was shown that LLIs lead to an increase in pelvic torsion, which is known to cause an anterior rotation of the hemipelvis on the side of the shorter leg [31]. Studies that use similar/equivalent imaging techniques to measure the effects of LLIs do support the findings of our study, regarding the effects of LLIs.

In a recent study, simulated LLIs of > 1 cm caused a significant increase in lateral deviation and surface rotation of the spine [12]. Although, our groups differed on average more than 1.5 cm in their leg length inequalities, we did not find any differences in spinal position between the two patient groups, indicating that patients can even compensate LLIs > 2 cm without causing significant alterations in their spinal posture [2].

To the best of our knowledge, this is the first study to directly compare and evaluate two patient groups with LLIs smaller and greater than 2 cm. Previous studies have supported that LLIs ≥ 2 cm need to be treated clinically since they can lead to back and hip pain and to an increased risk in knee and hip osteoarthritis [1–3, 8]. Contrary to these findings, our results show that the group with LLIs > 2 cm only differed in pelvic obliquity and no other differences were found. These results are clinically meaningful and relevant as it raises the question on the necessity of the treatment of LLIs of two and more centimeters.

Further comparison of the patient groups under dynamic conditions, revealed almost no significant differences for pelvic obliquity throughout the gait cycle and no significant differences for the spinal parameters. These findings support earlier work with simulated LLIs, that demonstrated greater compensation of LLIs during walking [4, 6, 12]. For our walking trials we found a significant difference between the two groups for pelvic obliquity during the midstance phase of the longer leg. The different compensation strategies for pelvic obliquity of patients with LLIs while walking were previously confirmed by Song et al. who analysed a collective of 35 children with various LLIs (0.6–11.1 cm). Their study showed that only two patients presented with increased pelvic obliquity, which was not correlated with the degree of limb-length discrepancy. The authors stated that patients with LLIs and non-relevant co-morbidities are likely to develop various compensation strategies of the lower limb to compensate for the LLIs while walking [32]. Kakushima et al. simulated LLIs of 3 cm and found a significant increase in lateral bending of the spine [33], while Needham et al., identified only minimal differences in pelvic and spinal motion in subjects with simulated LLIs [34]. These studies again confirm that there must exist various compensation strategies in different cohorts of subjects with LLIs, highlighting the fact that a more individualized approach might be necessary to understand and examine the effects of LLIs on the body.

There do exist some limitations of this present study that need to be addressed. The present investigation does focus on the biomechanical effects of LLIs on the spine and pelvis. However, it does not allow to compare the effects of LLIs on the ankle, knee and hip joint due to the type of imaging system chosen, which could also be clinically relevant. Further studies should therefore include measurements of the lower extremities in order to examine the role of the lower extremities on the compensation of LLIs.

We have to acknowledge that we only measured the patients´ LLIs once and did not perform repeated measurements. However, based on several studies the single measurement by a single expert examiner provides good and sufficient reliability [19, 20, 35].

It has been shown good correlation to CT (ICC: 0.805) and a second examiner (ICC: 0.92) by Jamaluddin et al. while an older study by Beattie et al. revealed an ICC of 0.770 compared to conventional radiographs [19, 35]. Neely et al. reported even excellent reliability of single tape measurement of leg length by two independent single examiners compared to CT with ICC: 0.984 for examiner 1 and 0.978 for examiner 2 [20].

Another limitation is the relatively small number of patients included and the heterogeneity of the aetiology of the LLIs, which may not allow to generalize our findings.

A review article by Davis et al. pointed out that patient populations regarding LLI are difficult to control in terms of their origin, localization, cause or amount [2]. Studies on patients with real LLIs are rare over multiple evaluations on simulated LLIs. Those who evaluated the effect of LLIs on gait by Aiona et al., and Song et al. and Pertunnen et al. examined patients with both acquired and congenital leg length discrepancies [5, 32, 36]. While studies do not differentiate in this matter specific compensation mechanisms or differences are not known or evaluated [2]. In our study patients with congenital and acquired LLIs were included.

Further, we did not compare our two patient groups with a control group, which would have allowed a more comprehensive analysis of the effects of LLIs.

However, the primary focus of this pilot study was to compare patients with LLIs of a certain extend to each other biomechanically. In future studies, we intend to evaluate a greater patient collective with separated populations of acquired and congenital LLIs and an additional comparison to a healthy control group for further clarification regarding the amount of LLI that requires treatment.

## Conclusions

The results of our study suggest that relevant LLIs of ≥ 2 cm mostly affect pelvic obliquity and do not lead to significant alterations in the spinal posture during a standing trial. Additionally, we demonstrated that LLIs are better compensated when walking, showing almost no significant differences in pelvic and spinal posture between patients with LLIs smaller and greater than 2 cm. This study shows that even LLIs ≥ 2 cm can be compensated in the lower extremities and therefore no differences on the pelvic and spinal level occured; however, we do not know if the compensation mechanisms may lead to long-term clinical symptoms and pathologies. These findings raise the question, which LLIs need to be clinically treated and which can still be compensated for by the patient?

## Data Availability

The data that support the findings of this study are available from Marcel Betsch and Roman Michalik, but restrictions apply to the availability of these data, which were used under license for the current study, and so are not publicly available.
